# Prevalence and acquisition rate of extended spectrum beta lactamase producing gram-negative organisms (ESBL-GNO) in general medical patients in Switzerland

**DOI:** 10.1186/1753-6561-5-S6-O24

**Published:** 2011-06-29

**Authors:** J Pasricha, T Koessler, V Camus, S Harbarth, G Renzi, J Schrenzel, D Pittet, A Perrier, A Iten

**Affiliations:** 1 Infection Control, University of Geneva Hospital, Geneva, Switzerland; 2 Internal Medicine, University of Geneva Hospital, Geneva, Switzerland; 3 University of Geneva Hospital, Geneva, Switzerland; 4 Central Laboratory Bacteriology, University of Geneva Hospital, Geneva, Switzerland

## Introduction / objectives

We aimed to determine the prevalence and acquisition rate of ESBL-GNO amongst patients admitted to general medical units at our hospital.

## Methods

Patients consecutively admitted to 13 medical wards from March-June 2010 were screened for ESBL-GNO via rectal swab within 48hours of admission, and 36 hours of discharge.

## Results

Of 1967 patients, swabs were obtained in 1111(56%) at admission and 491(25%) at discharge with 441(22%) having both. Mean age was 64 years and 58% were male. 6.8% (75/1111) of patients were positive for an ESBL-GNO at admission of whom 86%, for whom data were available (24/28), had an ESBL-GNO detected in the previous 6 months. 3.7%(18/487) of patients acquired an ESBL-GNO, having positive cultures at discharge but not at admission. On univariate regression, acquisition of an ESBL-GNO was associated with admission from home (OR 0.2 [95%CI 0.1-0.6], p=.005), transfer from another unit (OR 8.3[95% CI 2-33], p=.003) and receipt of a first or second-generation cephalosporin (OR 7.2 [95% CI 1-37],p=.017). Receipt of a first or second-generation cephalosporin was the only factor independently associated with ESBL-GNO acquisition (OR 7.1[95% CI 1-40], p=.03). Age, sex, intensive care, provenance and receipt of other antibiotics were not associated with ESBL carriage or acquisition.

## Conclusion

Carriage and acquisition of ESBL-GNO is a problem amongst medical patients at our hospital. No risk factors for ESBL-GNO carriage were identified.

## Disclosure of interest

None declared.

